# The impact of 22q11.2 deletion syndrome on caregivers: assessing quality of life and burden

**DOI:** 10.1186/s13023-025-03790-5

**Published:** 2025-06-01

**Authors:** Dariusz Walkowiak, Jan Domaradzki

**Affiliations:** 1 Department of Organization and Management in Health Care, Poznan University of Medical Sciences, św. Marii Magdaleny 14, 61-786 Poznan, Poland; 2 Department of Social Sciences and Humanities, Poznan University of Medical Sciences, Poznan, Poland

**Keywords:** 22q11.2DS, Caregivers, Rare diseases, WHO Quality of Life-BREF, Zarit Burden Interview, Diagnostic odyssey

## Abstract

**Background:**

Raising a child with 22q11.2DS presents significant psychosocial and financial challenges for caregivers for various reasons. Research on the quality of life (QoL) of caregivers for patients with rare diseases highlights significant challenges, with caregivers reporting lower health-related QoL compared to the general population. Long-term care impacts social, health, and economic aspects of life, with the burden on caregivers inversely correlating with their QoL, affecting mobility and daily activities. Parents often manage their child's complex medical care, underscoring the need for comprehensive support systems. An online survey was developed to examine the QoL and burden of caregivers for patients with 22q11.2DS. The study utilized two tools: the Polish version of the World Health Organization (WHO) Quality of Life-BREF and the Polish version of the Zarit Burden Interview.

**Results:**

The survey was completed by 134 Polish caregivers, including 125 women, with 52 not employed due to caregiving for 22q11.2DS patients. Financial security and expenditure ability were found to significantly impact all four WHOQOL Domains, with financial well-being emerging as the primary factor. Although other factors influence each Domain, financial well-being is key. The financial factor also appears in caregiver burden analysis, though not as the main influence on burden. Most caregivers rated their quality of life (QoL) significantly below the general population average: 85.1% reported below-average QoL in the Physical Domain, and 83.6% in the Social Relationships Domain. The study highlights strong correlations between WHOQOL Domains, suggesting substantial interconnections. Professional activity was shown to positively impact the Psychological Domain QoL and is crucial for reducing caregiver burden. Additionally, diagnostic delays continue to affect caregivers’ QoL negatively.

**Conclusion:**

Efforts must be intensified to develop an efficient and rapid diagnostic system for 22q11.2DS. A comprehensive and holistic care system should be established to provide early and integrated support as soon as possible after birth. Psychological support for caregivers is essential, including both psychological and psychiatric assistance, respite care, and support from social institutions. This support should aim to relieve caregivers, allowing them time for themselves and the opportunity to pursue professional work if desired. Such measures can prevent feelings of exclusion and the perception that, despite being central to the care system for 22q11.2DS patients, their emotional and psychological needs are neglected by decision-makers. Targeted financial support should also be considered where necessary.

## Introduction

Chromosome 22q11.2 deletion syndrome (22q11.2DS) is the most frequent microdeletion syndrome, affecting 1 in 2000 to 4000 live births [1–4]. This rare multisystem genetic disorder presents a wide range of phenotypic expressions, from severe, life-threatening conditions to milder traits, and its manifestations are highly variable. Common medical issues in childhood include congenital heart defects, particularly conotruncal anomalies, palatal abnormalities such as velopharyngeal incompetence, immunodeficiency, hypocalcemia due to hypoparathyroidism, genitourinary anomalies, severe feeding and gastrointestinal issues, and subtle dysmorphic facial features. Neuropsychiatric disorders are also prevalent, with affected individuals at high risk for schizophrenia, intellectual disability, attention-deficit hyperactivity disorder (ADHD), autism spectrum disorder (ASD), anxiety and mood disorders, seizures, and early-onset Parkinson's disease [1, 2, 5–12]. The syndrome is linked to over 200 characteristics that can manifest in various combinations and levels of severity [13]. Mortality rates range from 5 to 15% in children, with most deaths occurring in the first year of life, and there is significant multimorbidity among those with 22q11.2DS [14]. The interplay between the syndrome's complexities and the challenges of navigating health services influences the standard of living of people with 22q11.2DS and their caregivers [15].

Despite 90% of individuals with 22q11.2DS having the same deleted region, the neuropsychiatric outcomes vary greatly across individuals and over the lifespan [16]. The genotype–phenotype relationship is not fully understood, with variability potentially due to genetic background effects, additional rare pathogenic mutations, and regulatory roles for certain genes within the deleted region. Genetic analysis is essential for definitive diagnosis [12, 16]. While most cases of 22q11.2DS are de novo, 8–28% of cases exhibit autosomal dominant inheritance [17].

The neurocognitive profile of individuals with 22q11.2DS is also highly variable, both within individuals and throughout development. Motor delays, sometimes with hypotonia, and speech-language difficulties are common from infancy. Learning challenges are typical in preschool and elementary school children [2, 18, 19]. The IQ distribution among children and teenagers with 22q11.2DS mirrors the general population but is shifted approximately 2 standard deviations to the left [20]. About one-third have mild to moderate intellectual disability, with the remainder falling in the borderline range (IQ 70–84) [2], and a decline in verbal IQ over time is common [12, 21, 22]. Most children with 22q11.2DS exhibit impaired language skills. Neurodevelopmental disorders, including ADHD (up to 40%) and ASD (up to 30%), are prevalent, necessitating periodic formal neuropsychological assessments. Anxiety disorders affect approximately 35% of children, with specific phobia, social phobia, and generalized anxiety disorder being most common. Severe intellectual disability is rare in children and adolescents but more frequent in adults, often linked to secondary insults or congenital brain malformations [1, 5, 12, 23, 24].

Individuals with 22q11.2DS face significant challenges in adapting to daily life [25]. These include difficulties in performing tasks like speaking due to palatal abnormalities and engaging in physical activities due to cardiac symptoms. Additional problems, such as integrating into school or the workplace, further impact their quality of life (QoL). Comprehensive pediatric care involving generalists and specialists is essential to address the broad spectrum of medical and developmental issues and their impact on well-being and QoL [1, 8, 12]. Understanding the variable expressivity, trait severity, temporal changes, and the importance of family-centered care is crucial.

Raising a child with 22q11.2DS imposes substantial psychosocial and financial burdens on family caregivers, which can affect their ability to care for the child. While several studies have assessed the QoL in caregivers of children with 22q11.2DS, few have examined the factors influencing QoL and caregiver burden [25, 26]. Understanding these factors is vital for providing support to caregivers and their families, ultimately benefiting children with 22q11.2DS. This study aims to identify and explain the factors affecting QoL and burden among Polish caregivers of patients with 22q11.2DS.

## Materials and methods

### Study design

As part of a larger project investigating the experiences of family caregivers of individuals with rare diseases (RDs) in Poland, this study was conducted as an anonymized, self-administered, quantitative web-based survey focusing on the quality of life (QoL) and caregiving burden of caregivers for those with 22q11.2DS. While the overall project aimed to understand the daily challenges faced by caregivers of 22q11.2DS patients, this particular survey concentrated on the caregivers’ self-reported QoL, perceived caregiving burden, and financial well-being.

Caregivers of individuals with 22q11.2DS were invited to participate. Recruitment was facilitated by the Polish 22q11 Association (Stowarzyszenie 22q11 Polska; https://www.22q11.pl/) from March to June 2024 through their social media platforms and newsletter, which included an invitation letter with a brief description of the research team, study aims, methods, and a link to the online questionnaire. All participants provided written informed consent online before completing the survey. Two follow-up invitations were sent in April and June.

Ethics and research governance approval were obtained from the Poznan University of Medical Sciences Bioethics Committee (KB–228/24). All study procedures conformed to the Declaration of Helsinki, and participants provided written informed consent prior to survey completion [27]. Consent was obtained before participants began filling out the questionnaire, and without it, participation in the study was not possible. Respondents were informed that they could withdraw from the study at any time and for any reason, without any consequences.

### Subjects

Participants were recruited via the Facebook page of the Polish 22q11 Association. This study utilized convenience sampling, and eligible participants were adults, born and residing in Poland at the time of the survey. They needed to be a parent or family caregiver of a person with a confirmed 22q11.2DS diagnosis, capable of using electronic devices to participate in an online survey, and able to provide written informed consent.

### Research instruments

The survey questionnaire consisted of four sections. The first section included an original ad hoc questionnaire with eight single-choice, closed-ended items concerning caregivers’ socio-demographic characteristics. The remaining sections comprised three standardized research tools to measure caregivers’ QoL, perceived burden, and financial well-being.

To assess caregivers’ QoL, the abbreviated Polish version of the World Health Organization (WHO) Quality of Life-BREF(WHOQOL-BREF) was used [28, 29]. This instrument, widely used in epidemiological studies and clinical trials, includes 24 items divided into Domains of physical health (7 items, raw score range: 7–35), psychological health (6 items, raw score range: 6–30), social relationships (3 items, raw score range: 3–15), and environmental health (8 items, raw score range: 8–40). Additionally, it includes two items on the individual’s overall perception of QoL and health satisfaction. Respondents rate each item based on their experiences over the previous two weeks using a Likert scale from 1 to 5, with higher scores indicating better QoL.

To measure the perceived burden of 22q11.2DS caregivers, we used the Polish version of the Zarit Burden Interview (ZBI) [30]. This self-administered scale comprises 22 items assessing the impact on psychological well-being, financial situation, caregiver-care recipient relationship, and social life. Each item is scored on a 5-point Likert scale, with a total score ranging from 0 to 88. Higher scores indicate greater caregiving burden.

For financial well-being assessment, we utilized the CFPB Financial Well-Being Scale (CFPB-FWBS) developed by the Consumer Financial Protection Bureau (CFPB) [31]. This free tool includes 10 questions evaluating an individual’s financial security and freedom of choice. Each item is scored from 0 to 4, with a total score ranging from 0 to 100 and 50 as the midpoint. Since there is no Polish version of the CFPB FWBS, it was back-translated by two experts proficient in English and Polish and adapted to Polish conditions. It was then pre-tested on two groups of 20 individuals: one group of caregivers for children with chronic diseases and another group of healthy individuals without additional family or financial burdens. The tool demonstrated high consistency and sensitivity, even in small sample sizes.

### Statistical analysis

Sociodemographic data are presented in two tables, including the age of 22q11.2DS patients as medians, means, and standard deviations. Other data are reported as the frequency of responses and the percentage of all responses. Results from the WHOQOL, ZBI, and CFPB-FWBS are presented with range, medians, means, and standard deviations. We fitted five logistic regression models to test for differences in QoL of our respondents across the four WHOQOL Domains and caregiver burden measured by the ZBI. National average values were used as the cut-off points in the WHOQOL Domains, while a ZBI score of 41 was considered indicative of burden. The explanatory variables were all sociodemographic data from Tables 1 and 2, with the age of the patient with 22q11.2DS and the CFPB-FWBS result as covariates. Due to the small sizes of some subgroups in the sociodemographic data, they were consolidated into two groups: individuals with higher education vs. others, employed vs. unemployed, those living in cities with up to 100,000 inhabitants vs. those in larger cities, diagnosis time up to one year vs. over one year, and patients correctly diagnosed the first time vs. those who received an accurate diagnosis only after one or more incorrect ones. Step-wise regression was used with entry and removal criteria set at *p* < 0.05 and *p* > 0.10, respectively. Spearman's correlations were used to measure associations between WHOQOL Domains and the ZBI. A significance level of 5% was adopted. All analyses were conducted in JASP 0.18.3.

**Table 1:** Socio-demographic characteristics of 22q11.2DS caregivers’

Characteristics	N (%)
*Sex*	
woman	125 (93.3)
man	9 (6.7)
*Age*	
16–19	1 (0.7)
20–29	6 (4.5)
30–39	58 (43.3)
40–49	59 (44)
50–59	7 (5.2)
60–69	3 (2.2)
*Education*	
primary school	2 (1.5)
vocational school	18 (13.4)
high school	26 (19.4)
university	82 (61.2)
medical university	6 (4.5)
*Domicile*	
up to 10,000 inhabitants	50 (37.3)
10–50,000 inhabitants	26 (19.4)
51–100,000 inhabitants	14 (10.4)
101–500,000 inhabitants	19 (14.2)
above 500,000 inhabitants	25 (18.7)
*Professional activity*	
unemployed	1 (0.7)
unemployed due to childcare	52 (38.8)
employed part-time	12 (9)
employed full-time	65 (48.5)
pension	4 (3)
*What role does religion play in your life?*	
significant	26 (19.4)
rather significant	36 (26.9)
little	40 (29.9)
none	32 (23.9)
*Relationship with a 22q11.2DS child*	
mother	122 (91)
father	9 (6.7)
sister	1 (0.7)
legal guardian	2 (1.5)

The Cronbach’s alpha values estimated for the various Domains were as follows: WHOQOL Physical Domain at 0.807, 95% CI [0.751–0.853], WHOQOL Psychological Domain at 0.793, 95% CI [0.733–0.842], WHOQOL Social Relationships Domain at 0.714, 95% CI [0.617–0.789], WHOQOL Environment Domain at 0.836, 95% CI [0.789–0.874], ZBI at 0.923, 95% CI [0.903–0.940], and CFPB-FWBS at 0.924, 95% CI [0.903–0.941]. While the Cronbach’s alpha estimate for the WHOQOL Social Relationships Domain is relatively low, it is important to note that this Domain consists of only three questions. Nonetheless, the score obtained for our study group was higher than that found in the validation process of this scale in Poland [29].

## Results

Table 1 presents the socio-demographic characteristics of caregivers of individuals with 22q11.2DS. The majority of caregivers were women (93.3%) and primarily fall within the age range of 30–49 years (87.3%). The majority of caregivers were mothers (91%) of the affected individuals. Educational backgrounds vary, with a significant proportion having university degrees (65.7%). Caregivers were distributed across different domicile sizes, with the highest percentage living in areas with up to 10,000 inhabitants (37.3%). In terms of professional activity, nearly half were employed full-time (48.5%), while a significant portion were unemployed due to childcare responsibilities (38.8%). Religious significance in caregivers' lives is mixed, with many considering it of little (29.9%) or no importance (23.9%).

Table 2 outlines the characteristics of patients with 22q11.2DS. Among the patients, 43.3% were female and 56.7% were male. The ages range from 0.4 to 38 years, with a mean age of 9.9 years (SD = 6.8) and a median age of 9.8 years. The time taken to obtain a diagnosis from the first symptoms varies widely: 46.3% were diagnosed within 6 months, while others took much longer, with 14.2% taking over 6 years. Most patients (70.1%) did not receive any previous diagnoses before the correct diagnosis was made, while 11.9% had one previous diagnosis, and smaller percentages had between 2 and 10 previous diagnoses.

**Table 2:** 22q11.2DS patient’s characteristics

Characteristics	N (%)
*Sex*	
female	58 (43.3)
male	76 (56.7)
*Age (in years)*	
Range	0.4–38
Mean (SD)	9.9 (6.8)
Median	9.8
*How long did it take to obtain a diagnosis from first symptoms?*	
1 month	39 (29.1)
2–3 months	17 (12.7)
4–6 months	6 (4.5)
7–12 months	17 (12.7)
1–2 years	15 (11.2)
3–5 years	21 (12.7)
6–7 years	6 (4.5)
8–10 years	6 (4.5)
11–15 years	4 (3)
16–20 years	2 (1.5)
over 20 years	1 (0.7)
*Number of previous diagnoses before the correct diagnosis was made*	
0	94 (70.1)
1	16 (11.9)
2–3	15 (11.2)
4–5	4 (3)
6–7	2 (1.5)
8–10	3 (2.2)

Table 3 summarizes descriptive statistics for quality of life and financial well-being scales. The majority of respondents scored below the national average across all WHOQOL domains, particularly in the Physical (85.1%) and Social Relationships (83.6%) domains. Mean scores ranged from 50.5 (Social Relationships) to 56.9 (Environment). Caregiver burden, as measured by the ZBI, averaged 37.1, while the CFPB-FWBS had a mean of 51.8, reflecting respondents’ perceived financial well-being.

**Table 3:** Descriptive statistics for WHOQOL domains, ZBI and CFPB-FWBS

Scale	Descriptive statistic	Number of respondents with score below mean country score N (%)
*WHOQOL Physical Domain*		
Range	13–94	114 (85.1)
Mean (SD)	53.9(17.9)	
Median	56	
*WHOQOL Psychological Domain*		
Range	13–94	93 (69.4)
Mean (SD)	56.2 (16.9)	
Median	56	
*WHOQOL Social Relationships Domain*		
Range	0–100	112 (83.6)
Mean (SD)	50.5 (20.2)	
Median	50	
*WHOQOL Environment Domain*		
Range	19–94	86 (64.2)
Mean (SD)	56.9 (17)	
Median	56	
*ZBI*		
Range	8–76	
Mean (SD)	37.1(14.8)	
Median	37.5	
*CFPB-FWBS*		
Range	27–81	
Mean (SD)	51.8 (12.2)	
Median	51.5	

Table 4 presents the results of a stepwise logistic regression analysis examining factors influencing the WHOQOL Physical Domain. Across all models, the CFPB-FWBS financial well-being score shows a statistically significant and positive association with physical quality of life. As additional variables are introduced, Domicile demonstrates a significant negative impact. Time to Diagnosis shows a negative association that becomes statistically significant in later models. The Age of the 22q11 patient has a positive influence on physical quality of life. Higher Education, included in the final model, also shows a positive association, though marginally significant. The findings suggest that greater financial well-being, quicker diagnosis, older patient age, and higher education are associated with better physical functioning, whereas less favorable residential location is linked to poorer physical outcomes.

**Table 4:** Stepwise logistic regression analysis of WHOQOL Physical Domain

Parameter	Estimate	Standard error	Odds Ratio	Wald test		
				Wald statistic	df	*p*
Model 1						
Intercept	− 1.740	0.242	0.175	51.542	1	< 0.001
Model 2						
Intercept	-9.487	1.956	7.585 × 10^–5^	23.526	1	< 0.001
CFPB-FWBS	0.135	0.032	1.145	18.157	1	< 0.001
Model 3						
Intercept	− 9.610	1.949	6.702 × 10^–5^	24.303	1	< 0.001
CFPB-FWBS	0.145	0.032	1.156	20.168	1	< 0.001
domicile	− 1.486	0.691	0.226	4.623	1	0.032
Model 4						
Intercept	− 9.561	2.010	7.039 × 10^–5^	22.619	1	< 0.001
CFPB-FWBS	0.149	0.033	1.161	19.981	1	< 0.001
domicile	− 1.366	0.698	0.255	3.832	1	0.050
time to diagnosis	− 1.326	0.734	0.266	3.260	1	0.071
Model 5						
Intercept	− 11.691	2.503	8.370 × 10^–6^	− 4.671	1	< 0.001
CFPB-FWBS	0.171	0.038	1.186	4.500	1	< 0.001
domicile	− 1.481	0.716	0.227	− 2.069	1	0.039
time to diagnosis	− 2.252	0.907	0.105	− 2.483	1	0.013
age of 22q11 patient	0.112	0.053	1.119	2.102	1	0.036
Model 6						
Intercept	− 12.853	2.794	2.619 × 10^–6^	21.157	1	< 0.001
CFPB-FWBS	0.163	0.039	1.177	17.336	1	< 0.001
domicile	− 1.575	0.722	0.207	4.755	1	0.029
time to diagnosis	− 2.357	0.937	0.095	6.330	1	0.012
age of 22q11 patient	0.143	0.060	1.154	5.613	1	0.018
higher education	1.781	0.908	5.936	3.843	1	0.050
Model summary						
2. *p* < 0.001, ΔΧ^2^ = 28.808, Nagelkerke R^2^ = 0.340						
3. *p* = 0.019, ΔΧ^2^ = 5.475, Nagelkerke R^2^ = 0.396						
4. *p* = 0.052, ΔΧ^2^ = 3.775, Nagelkerke R^2^ = 0.434						
5. *p* = 0.033, ΔΧ^2^ = 4.529, Nagelkerke R^2^ = 0.478						
6. *p* = 0.030, ΔΧ^2^ = 4.719,, Nagelkerke R^2^ = 0.522						

Table 5 presents the stepwise logistic regression results for the WHOQOL Psychological Domain. Initially, only the intercept is included and found to be significant. In the second model, adding the CFPB-FWBS financial well-being score reveals a significant positive association with psychological quality of life, and the model’s overall fit improves notably. In the third model, Professional Activity is added and also shows a significant positive relationship. Each step improves the model’s explanatory power, with both financial well-being and professional activity emerging as significant predictors of better psychological functioning.

**Table 5:** Stepwise logistic regression analysis of WHOQOL Psychological Domain

Parameter	Estimate	Standard error	Odds Ratio	Wald Test		
				Wald statistic	df	*p*
Model 1						
Intercept	− 0.819	0.187	0.441	19.088	1	< 0.001
Model 2						
Intercept	− 8.089	1.466	3.070×10^–4^	30.434	1	< 0.001
CFPB-FWBS	0.133	0.026	1.142	26.983	1	< 0.001
Model 3						
Intercept	− 8.421	1.524	2.201×10^–4^	30.550	1	< 0.001
CFPB-FWBS	0.124	0.026	1.132	23.512	1	< 0.001
professional activity	1.158	0.525	3.183	4.866	1	0.027
Model summary						
2. *p* < 0.001, ΔΧ^2^ = 43.767, Nagelkerke R^2^ = 0.393						
3. *p* = 0.022, ΔΧ^2^ = 5.212,, Nagelkerke R^2^ = 0.432						

Table 6 shows the stepwise logistic regression analysis for the WHOQOL Social Relationships Domain. The first model includes only the intercept, which is highly significant. In the second model, the CFPB-FWBS financial well-being score is added and shows a significant positive relationship with social quality of life, improving the model’s fit. In the third model, the Number of Diagnoses is included and reveals a significant negative association, indicating that a higher number of diagnoses is linked to lower social well-being. Both financial well-being and the number of diagnoses significantly influence the quality of social relationships, with each model showing improved explanatory power.

**Table 6:** Stepwise logistic regression analysis of WHOQOL Social Relationships Domain

Parameter	Estimate	Standard error	Odds Ratio	Wald test		
				Wald statistic	df	*p*
Model 1						
Intercept	− 1.627	0.233	0.196	48.703	1	< 0.001
Model 2						
Intercept	− 4.410	1.202	0.012	13.470	1	< 0.001
CFPB-FWBS	0.051	0.021	1.053	6.056	1	0.014
Model 3						
Intercept	− 4.113	1.255	0.016	10.743	1	0.001
CFPB-FWBS	0.052	0.022	1.053	5.563	1	0.018
number of diagnosis	− 1.609	0.780	0.200	4.260	1	0.039
Model summary						
2. *p* = 0.01, ΔΧ^2^ = 6.588, Nagelkerke R^2^ = 0.081						
3. *p* = 0.015, ΔΧ^2^ = 5.926,, Nagelkerke R^2^ = 0.151						

Table 7 summarizes the stepwise logistic regression analysis for the WHOQOL Environment Domain. The first model includes only the intercept and is statistically significant. In the second model, the addition of the CFPB-FWBS financial well-being score significantly improves the model, showing a strong positive association with the environmental quality of life. In the third model, the Number of Diagnoses is added but does not significantly contribute to the model, although a slight improvement in explanatory power is observed. Overall, financial well-being remains the key factor positively influencing the environmental domain of quality of life.

**Table 7:** Stepwise logistic regression analysis of WHOQOL Environment Domain

Parameter	Estimate	Standard error	Odds Ratio	Wald test		
				Wald statistic	df	p
Model 1						
Intercept	− 0.583	0.180	0.558	10.476	1	0.001
Model 2						
Intercept	− 7.648	1.375	4.769×10^–4^	30.946	1	< 0.001
CFPB-FWBS	0.131	0.025	1.140	28.516	1	< 0.001
Model 3						
Intercept	− 7.519	1.396	5.426 × 10^–4^	28.990	1	< 0.001
CFPB-FWBS	0.132	0.025	1.141	27.820	1	< 0.001
number of diagnosis	− 0.748	0.510	0.473	2.154	1	0.142
Model summary						
2. *p* < 0.001, ΔΧ^2^ = 46.102, Nagelkerke R^2^ = 0.399						
3. *p* = 0.134, ΔΧ^2^ = 2.247, Nagelkerke R^2^ = 0.416						

Table 8 presents the results of a stepwise logistic regression analysis for the ZBI score. Initially, the model includes only the intercept, which is statistically significant. In Model 2, the inclusion of Professional Activity significantly improves the model, revealing that being professionally active is associated with lower caregiver burden. Model 3 adds Higher Education, which is positively associated with greater burden, and both predictors remain significant. In the final model, the CFPB-FWBS financial well-being score is introduced and shows a significant negative association with burden, indicating that higher financial well-being is linked to lower caregiver burden. Each model step enhances explanatory power, with Professional Activity, Higher Education, and CFPB-FWBS all contributing significantly.

**Table 8:** Stepwise logistic regression analysis of ZBI

Parameter	Estimate	Standard error	Odds Ratio	Wald test		
				Wald statistic	df	*p*
Model 1						
Intercept	− 0.362	0.176	0.696	4.252	1	0.039
Model 2						
Intercept	0.391	0.270	1.478	2.096	1	0.148
professional activity	− 1.372	0.372	0.254	13.598	1	< 0.001
Model 3						
Intercept	− 0.001	0.330	0.999	8.475 × 10^–6^	1	0.998
professional activity	− 1.786	0.444	0.168	16.166	1	< 0.001
higher education	0.947	0.466	2.578	4.137	1	0.042
Model 4						
Intercept	1.723	0.864	5.603	3.975	1	0.046
professional activity	− 1.658	0.456	0.191	13.213	1	< 0.001
higher education	1.149	0.492	3.154	5.456	1	0.020
CFPB-FWBS	− 0.038	0.017	0.963	4.679	1	0.031
Model summary						
2. *p* < 0.001, ΔΧ^2^ = 14.322, Nagelkerke R^2^ = 0.137						
3. *p* = 0.034, ΔΧ^2^ = 4.489,, Nagelkerke R^2^ = 0.177						
4. *p* = 0.027, ΔΧ^2^ = 4.862,, Nagelkerke R^2^ = 0.218						

Table 9 presents the Spearman’s correlation coefficients between the WHOQOL-BREF domains and the ZBI. Strong positive correlations are observed between the Physical and Psychological domains, as well as between Psychological and Environment, and Psychological and Social Relationships, indicating that better physical and psychological health are closely linked. Moderate to strong correlations also exist between other domains, such as Physical and Environment or Social Relationships and Environment. In contrast, all correlations between WHOQOL-BREF domains and ZBI are negative, meaning that higher quality of life scores are associated with lower caregiver burden. The strongest negative associations are between ZBI and the Physical and Psychological domains, while the Social Relationships and Environment domains show slightly weaker, but still significant, inverse relationships.

**Table 9:** Spearman’s correlations between WHOQOL-BREF Domains and ZBI

	Spearman’s rho	Lower 95% CI	Upper95% CI	*p*
Physical-Psychological	0.744	0.657	0.811	< 0.001
Physical-Social Relationships	0.568	0.441	0.673	< 0.001
Physical-Environment	0.676	0.571	0.758	< 0.001
Psychological-Social Relationships	0.724	0.632	0.796	< 0.001
Psychological-Environment	0.745	0.659	0.812	< 0.001
Social Relationships- Environment	0.651	0.541	0.739	< 0.001
Physical-ZBI	− 0.570	− 0.674	− 0.443	< 0.001
Psychological-ZBI	− 0.551	− 0.659	− 0.420	< 0.001
Social Relationships-ZBI	− 0.382	− 0.518	− 0.227	< 0.001
Environment-ZBI	− 0.400	− 0.533	− 0.247	< 0.001

## Discussion

Research on the quality of life of caregivers for RDs patients reveals significant challenges. Caregivers consistently report lower health-related quality of life compared to the general population [32–35]. Long-term care for a child with a serious, incurable disease leads to substantial changes in social, partner, health, and economic aspects of life [36, 37]. The burden on caregivers is inversely correlated with their quality of life, affecting their mobility, daily activities, and levels of pain and discomfort [38]. Parents of children with genetic disorders face the dual challenge of managing day-to-day issues while worrying about their children's current functioning and future ability to lead fulfilling adult lives. It has been reported that parents often feel responsible for managing their child's complex and fragmented medical care [25, 39]. Factors such as patient age and the time devoted to care significantly impact various dimensions of caregiver quality of life [40, 41]. These findings underscore the need for comprehensive support systems and interventions to address the unique challenges faced by caregivers of rare disease patients [42]. Given that 22q11.2DS is one of the more prevalent rare diseases, our study is particularly relevant to a substantial group of individuals.

Financial burden is a major challenge for caregivers of patients with RDs. The economic costs families must cover often force caregivers to deplete their savings. Purchasing specialized equipment, hiring professionals, and meeting additional financial demands associated with RDs place significant stress on family life. These pressures can hinder a caregiver’s ability to address the patient’s healthcare needs, further increasing their strain [25, 38]. Beyond direct healthcare costs, caregivers often report that their career choices are affected by caregiving responsibilities, a trend research suggests primarily impacts women [34]. Many reduce working hours or leave their jobs altogether, leading to lost income and financial insecurity. Much of the diagnostic process, patient therapy, and psychological support for caregivers is paid out of pocket, often outside the healthcare system. Many caregivers give up professional careers to care for their children, and later, adult patients who may still require care [43–45].

Our study shows that the subjective sense of financial security and the ability to make various expenditures, including those unrelated to the disease, significantly impact all four WHOQOL domains. We could not confirm the old saying that money cannot buy happiness, as this was not supported by our findings among caregivers for patients with 22q11.2DS. CFPB-FWBS emerged as the strongest influence across all domains. While logistic regression revealed other contributing factors, financial well-being appears to be the key one. Additionally, in the caregiver burden analysis, the financial factor reappears, though it is not the primary influence on burden perception.

Our study demonstrated that the vast majority of caregivers rate their QoL significantly below the average value reported by their statistical compatriots. Specifically, 85.1% of our respondents rated their QoL below mean in the Physical Domain, and 83.6% did so in the Social Relationships Domain. In a recent study by Silva and Gil-da-Silva-Lopes [26], which also used the same tool to examine the QoL of Brazilian caregivers of patients with 22q11.2DS, the results differed from those of the control group except in the Social Relationships Domain. However, it appears that the QoL scores of Polish caregivers were lower than those observed in the Brazilian study.

Barros et al. [46] obtained similar results using the WHOQOL-BREF in a group of primary caregivers of children and young adults with disabilities, where QoL in all four Domains differed from the control group. Guarany et al. [47] also reported similar findings in mothers of patients with mucopolysaccharidosis, with the physical health Domain scoring highest and the environmental Domain scoring lowest. Kim's study [48] on mothers of patients with mitochondrial disease showed reduced QoL in the Physical and Psychological Domains. In caregivers of children and adolescents with intellectual disabilities [49], QoL mean scores were lower than the general population across all four Domains. Similarly, caregivers of children with phenylketonuria also reported lower QoL mean scores in all four Domains compared to the general population [50]. In a study on parents of children with inborn errors of metabolism [51], parental QoL was positively impacted in terms of psychological health but negatively impacted in physical health and social relationships compared with the age- and sex-matched general population.

Parents of children with RDs are known to be at risk of impaired QoL. However, the research results we cited above show that even when using identical tools, the outcomes are not entirely consistent. This inconsistency is likely due to cultural factors specific to different societies, the homogeneity of the groups studied, and consequently, varying experiences in caring for the patient. Additionally, study groups are often small, which, although not surprising given the rarity of these diseases, can affect the accuracy and representativeness of the results. In our study, the groups are homogeneous, consisting solely of caregivers of patients with 22q11.2DS, and large enough to allow for statistically significant comparisons.

Our study sheds new light on the internal relationships between individual WHOQOL Domains. While it is not surprising that various aspects of quality of life are interconnected, the high level of correlation between the Physical Domain and the Psychological and Environmental Domains, as well as between the latter two Domains, is noteworthy. This suggests a strong interpenetration of these Domains for many caregivers, indicating their mutual impact and interactions. Consequently, it may be beneficial to treat these Domains comprehensively and to be aware that deterioration or improvement in one specific area can affect the others.

The impact of professional activity on the well-being of caregivers for patients with rare diseases (RD) has been previously described [52, 53]. The current study demonstrates a direct impact of the caregiver's professional activity on their Psychological Domain QoL. Additionally, professional activity is a key factor in reducing burden associated with caregiving. Caring for someone with an RD presents a range of challenges influenced by severity and duration of the diseases, the evolving understanding of the condition, and the caregiver's ability to manage the emotional strain of extended caregiving [54, 55]. Caring for a patient with 22q11DS requires both extensive knowledge and significant sacrifices on many levels. Furthermore, as shown by Karas et al. [56], the scope of care during the patient's transition to adulthood changes, but psychiatric and behavioral issues are often perceived as the most challenging aspects of adulthood. It appears that even the most dedicated and loving caregivers are not automatons who continuously provide care without pause. They require moments of respite and social interaction, and from this perspective, professional employment can serve as a valuable break from the demands of around-the-clock caregiving. Furthermore, as indicated by our results, engaging in professional work contributes to the household income, thereby better addressing the needs of all household members, including the patient.

Our earlier study revealed that caregivers of 22q11DS patients face numerous challenges, only some of which are objective in nature [25]. Unfortunately, many issues stem from the lack of knowledge of healthcare professionals (HCPs) and the organization of the healthcare system, including the availability and funding of various medical procedures. A significant and persistent problem is the length of time required to obtain a diagnosis and the high number of specialists visited by parents before a diagnosis is made [25, 57–59]. In other studies, parents also reported feeling responsible for managing their child's complex and fragmented medical care. They expressed concerns about insufficient empathy and a lack of awareness of 22q11DS among HCPs [39]. The results of our current study confirm that diagnostic-related issues remain problematic despite the passage of time. The latest study shows that delays in diagnosing RDs are still a serious problem [60]. Moreover, our study highlights that the diagnosis process and its timing should not be viewed as a one-time event that, although unpleasant, ends without major consequences. From the caregiver's perspective, whether the diagnosis was prolonged or involved multiple inaccurate diagnoses, it impacts their QoL. This impact persists regardless of whether the prolonged diagnosis occurred a year ago or a decade ago. Caregivers who have experienced a delayed diagnosis of 22q11DS continue to experience a reduced quality of life.

## Limitations

The limitations of this research should be considered when interpreting the results. Firstly, the study included relatively small number of participants. However, it should be acknowledged that since there is no registry of patients with RDs in Poland, the exact number of individuals with 22q11.2DS is unknown. Secondly, since we reached patients’ caregivers primarily through the Polish 22q11 Association’s Facebook page there is a risk of recruitment bias since not all caregivers are associated. Due to the online nature of the survey, there is also a risk that mothers with limited access to the Internet were unable to participate and share their experiences. For both these reasons, the survey's findings reflect only the views of those caregivers who chose to participate and may not represent the entire population of 22q11.2DS caregivers in Poland. Thirdly, there is a possibility of misinterpretation of some phrases or questions because the survey is caregiver-reported. Fourthly, since only nine fathers completed the survey, the predominance of women among our respondents may introduce another bias. Thus, even though numerous studies confirm that there is a gender gap in caregiving, future studies should include perspective of male caregivers whose experiences can be different. Lastly, the study included only a limited number of questions regarding parents' perceptions of the patient's clinical condition, despite the primary focus being on caregivers' perceptions of the challenges associated with caregiving, focusing rather on experiences related to the diagnostic odyssey.

## Conclusions

Interventions aimed at improving caregivers' quality of life are crucial. Access to counseling and mental health services is essential for addressing the emotional and psychological challenges faced by caregivers. Financial assistance programs that offer support or help with medical costs can alleviate some of the financial burdens. Leveraging community resources, such as volunteer networks and local healthcare services, can provide additional support and relieve some of the caregiving burdens. In summary, the quality of life for caregivers of 22q11DS patients is often compromised by a combination of emotional, physical, financial, and social challenges. Addressing these issues requires a comprehensive support system that includes expediting the diagnostic process [61], providing emotional support [62] and respite care assistance, properly targeted financial aid, and practical caregiving resources.

## Data Availability

The data underlying this study’s findings cannot be shared publicly due to the sensitivity and privacy of the study participants. The data will be shared at a reasonable request by the corresponding author.

## Abbreviations

RDs: Rare diseases
22q11.2DS: 22Q11.2 deletion syndrome
ADHD: Attention-deficit hyperactivity disorder
ASD: Autism spectrum disorder
QoL: Quality of life
WHOQOL-BREF: World Health Organization quality of Life-BREF
ZBI: Zarit burden interview
CFPB-FWBS: Consumer financial protection bureau financial well-being scale
